# R213W mutation in the retinoschisis 1 gene causes X-linked juvenile retinoschisis in a large Chinese family

**Published:** 2010-08-12

**Authors:** Jun Xu, Hong Gu, Kai Ma, Xipu Liu, Torkel Snellingen, Erdan Sun, Ningli Wang, Ningpu Liu

**Affiliations:** 1 Beijing Tongren Eye Center, Beijing Tongren Hospital, Capital Medical University, Beijing Ophthalmology and Visual Sciences Key Laboratory, Beijing, China; 2 Sekwa Eye Hospital, Beijing, China

## Abstract

**Purpose:**

We identified a large Chinese family with X-linked juvenile retinoschisis. The purpose of this study was to report the clinical findings of the family and to identify the genetic mutation by screening the retinoschisis 1 (*RS1*) gene.

**Methods:**

Family history was collected and all family members underwent routine ophthalmic examination. Venous blood was collected from family members and genomic DNA was extracted. The exons of *RS1* were screened by PCR followed by direct sequencing and/or restriction enzyme digestion.

**Results:**

The pedigree of interest was a four-generation family with 52 family members, including seven affected individuals. The proband was a 5-year-old boy showing highly elevated bullous retinoschisis with moderate vitreous hemorrhage in both eyes. Vitrectomy was performed in the left eye of the proband. Five affected males showed large peripheral retinoschisis in both eyes, either involving the macula or combined with foveal stellate cystic change. One of the affected family members showed only a foveal stellate cystic change in both eyes without periphery retinoschisis. Visual acuity of affected individuals ranged from hand motion to 0.4. The R213W mutation in exon 6 of *RS1* was identified in all affected individuals, predicting an amino acid substitution of arginine to tryptophan at codon 213.

**Conclusions:**

Our data show that the R213W mutation in *RS1* causes various severities of retinoschisis in a large Chinese family, providing further evidence for X-linked juvenile retinoschisis phenotypic variability.

## Introduction

X-linked juvenile retinoschisis (XLRS) is a hereditary retinal disease characterized by a splitting of the neurosensory retina [1]. It is a major cause of juvenile retinal degeneration in males [2], with an estimated prevalence of 1 in 15,000 to 1 in 30,000 [3]. The typical clinical manifestations of XLRS include foveal stellate cystic changes and peripheral retinoschisis. Symptoms, including visual impairment, squint, or nystagmus, usually appear at age 4 to 8 years, and visual deterioration often progresses during the first to second decades of life [4]. Thereafter the disease is mainly stationary or slowly progressive. By the age of 40 to 50 years, macular degeneration often occurs, causing additional visual failure [5]. In addition, severe vision loss can come from secondary complications, such as vitreous hemorrhage, retinal detachment, and neovascular glaucoma. Female carriers of the disease are unaffected.

XLRS is caused by mutations in the retinoschisis 1 (*RS1*) gene localized at chromosome Xp22.2 [6]. The *RS1* gene has six exons and encodes a 224 amino acid protein called retinoschisin, which is primarily expressed in retina and reveals an important role in retinal cell adhesion [7]. More than 100 disease-causing mutations in *RS1* have been reported, including missense/nonsense mutations, deletions, insertions, and splice site mutations [8]. Only a few studies, however, have described the clinical features of families with defined mutations in *RS1* [9-17]. Here we report a large Chinese family in which a R213W missense mutation in *RS1* causes various degrees of severity of retinoschisis, providing evidence for phenotypic variability in XLRS.

## Methods

### The proband and the family

The proband was a 5-year-old boy. The family history was collected from his parents during their visit to the Beijing Tongren Hospital, Beijing, China. The proband, his parents, and his elder brother were examined during their visit to the hospital. All other family members were examined at their local residence in the countryside of southern China after informed consent was obtained. The study protocol was approved by the Ethics Committee of Beijing Tongren Hospital and conformed to the Declaration of Helsinki. Routine ophthalmic examination included visual acuity measured using a conventional Snellen chart, slit-lamp biomicroscopy, and direct and indirect ophthalmoscopy after pupil dilation. Digital color photographs of the fundus were obtained from the proband, his elder brother, and their parents. Color photography of the anterior segment and ultrasonography were also performed for the proband.

### Mutation screening

After informed consent for genetic testing was obtained, peripheral venous blood (3 ml) was collected from 28 family members in EDTA-containing tubes, stored at 4 °C, and transferred to −80 °C within 24 h. Genomic DNA was extracted from peripheral blood leukocytes using a genomic DNA extraction and purification kit (TIANamp Swab DNA Kit; TIANGEN Biotech, Beijing, China), according to the manufacturer’s protocol. Mutation screening was initially performed for the proband, his affected brother, and their parents, using a method of PCR followed by direct sequencing of the PCR products. Exons 1–6 of *RS1* were amplified by PCR, as previously described [6]. The PCR reaction was performed in a DNA thermocycler (Eppendorf, Hamburg, Germany) in a 20 μl mixture contains 2 μl 10× buffer (25 mM MgCl_2_), 0.25 mM each of dNTP, 10 pmol of each primer, 0.5 units of Taq DNA polymerase, and 50 ng of genomic DNA. PCR cycles for the *RS1* gene were as follows: 94 °C for 5 min, followed by 35 cycles of denature at 94 °C for 30 s, annealing for 30 s at the appropriate temperature for each primer pair as shown in Table 1, and extension at 72 °C for 45 s. The final extension step was lengthened to 5 min. The amplified products were resolved by electrophoresis in 2% (w/v) agarose gels with 0.5 μg/ml ethidium bromide and visualized under ultraviolet light. The PCR products were then directly sequenced using an automatic DNA analyzer (model 3730XL; Applied Biosystems, Inc., Foster City, CA). A comparison to a previously published wild-type reference sequence was made.

**Table 1:** Sequence of primers used to amplify the coding regions of the *RS1* gene

**Exon**	**Primers (5′-3′)**	**Size of amplified fragment (bp)**	**Annealing temperature (°C)**
1	CTCAGCCAAAGACCTAAGAAC	216	61
	GTATGCAATGAATGTCAATGG		
2	GTGATGCTGTTGGATTTCTC	176	59
	CAAAGTGATAGTCCTCTATG		
3	CTGCCCTGCCTCTCTGGTTG	178	59
	GGTGTTCCCAATGACTGTTCC		
4	GGTGCTTGTTGAGTATTGAG	219	62.5
	AAAATCCCCGGGCCCTGC		
5	GAGAGCCAGCACCTGCGG	311	65
	GGGTGCGAGCTGAAGTTGG		
6	CCCGATGTGATGGTGACAGG	414	58
	CTTTGTTCTGACTTTCTCTGGC		

This table summarizes the primer sequences, size of the amplified fragments and the annealing temperatures for each primer pairs.

Once the candidate mutation was identified, restriction enzyme MspI (5′…C^CGG…3′) recognizing the potential mutation site was used for all 28 family members with genomic DNA available. The PCR amplified products were digested with MspI at 37 °C for 5 h, according to the manufacturer’s protocol (New England Biolabs, Ipswich, MA). Samples were then electrophoresed on a 2% (W/V) agarose gel with 0.5 μg/ml ethidium bromide. Images of the gel were taken with a Molecular Imager Gel Doc XR System (Bio-RAD, Hercules, CA). Genotypes were determined based on the restriction patterns, which were further confirmed by sequencing of the PCR products with an automatic DNA analyzer in a selected subset of subjects.

## Results

### Clinical findings

**Figure 1:**
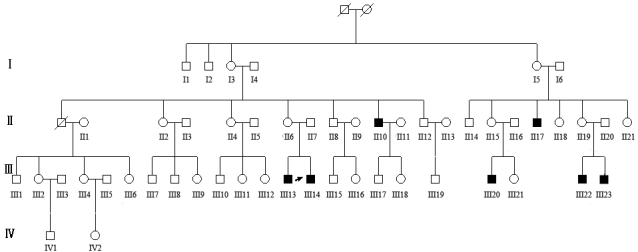
Pedigree of the Chinese family with X-linked juvenile retinoschisis. Squares represent males, and circles represent females. Solid symbols indicate affected subjects with X-linked juvenile retinoschisis (XLRS). Unfilled symbols represent unaffected family members. A diagonal line indicates a deceased family member. The arrow indicates the proband (III-14).

The pedigree of interest was a four-generation family with 52 family members, including 45 unaffected individuals and seven affected individuals based on clinical evaluation (Figure 1). An X-linked pattern of inheritance of disease was shown in the family pedigree. The age of onset, visual acuity, and peripheral and macular involvements for each affected individual are described in Table 2.

**Table 2:** Clinical findings of affected individuals in the Chinese family with X-linked juvenile retinoschisis

**Patient** **ID**	**Age of onset**	**Right eye**			**Left eye**		
**Visual acuity**	**Peripheral retinoschisis**	**Macular involvement**	**Visual acuity**	**Peripheral retinoschisis**	**Macular involvement**
II-10	7	FC	yes	MS	FC	yes	PRIM
II-17	8	0.4	no	MS	0.3	no	MS
III-13	4	0.1	yes	MS	0.05	yes	PRIM
III-14	2	0.05	yes	Unclear	HM	yes	MS
III-20	6	0.1	yes	PRIM	0.4	yes	MS
III-22	7	0.1	yes	PRIM	0.1	yes	PRIM
III-23	5	0.1	yes	MS	0.4	yes	PRIM

This table summarizes the clinical findings of 7 patients in the Chinese family with X-linked juvenile retinoschisis. Abbreviations: FC represents finger counting; HM represents hand motion; PRIM represents peripheral retinoschisis involving macula; MS represents macular schisis.

**Figure 2:**
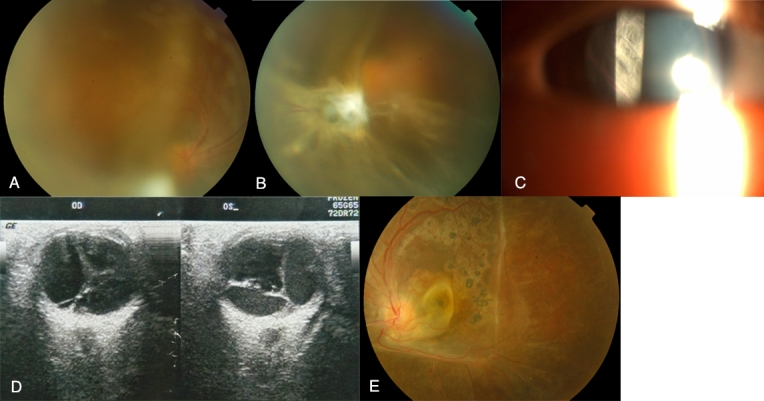
Photographs for the proband (III-14) of the family with X-linked juvenile retinoschisis. **A**: Fundus photograph of the right eye. **B**: Fundus photograph of the left eye. **C**: Slit-lamp photograph showing a membrane with blood vessels behind the lens. **D**: Ultrasonography of the right eye and left eye. **E**: Fundus photograph of the left eye after vitrectomy.

**Figure 3:**
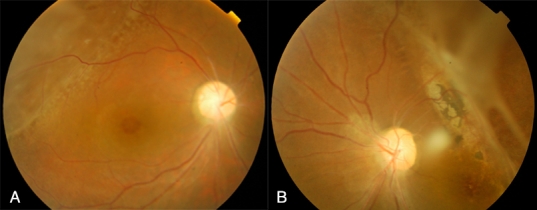
Fundus photographs for the elder brother (III-13) of the proband. Panel **A** describes the right eye showing a foveal stellate cystic change and a peripheral retinoschisis in the upper temporal retina. Panel **B** describes the left eye showing peripheral retinoschisis involving the macula.

The 5-year-old proband (III-14) was initially referred to the Beijing Tongren Hospital after progressive vision loss for the preceding 3 years and appearance of a “white pupil” in both eyes. On examination his visual acuity was 0.05 in the right eye and hand motion in the left eye. No evidence of squint or nystagmus was observed. Cornea, anterior chamber, iris, pupil, and lens were normal in both eyes. Severe vitreous scar tissue formation and moderate vitreous hemorrhage were noticed in both eyes (Figure 2A,B). On slit-lamp biomicroscopy a membrane with blood vessels was observed just behind the lens in his left eye (Figure 2C). Ultrasonography revealed echogenic bands in the vitreous extending from the posterior surface of the lens to the optic nerve in both eyes (Figure 2D). The elder brother (III-13) of the proband was a 7-year-old boy. His vision was noticed to be poor in both eyes when he was 4 years old. At the time of examination, his visual acuity was 0.1 in the right eye and 0.05 in the left. No squint or nystagmus was observed. Anterior segments of both eyes were normal. Fundus examination showed a foveal stellate cystic change and a large retinoschisis in the upper temporal retina of the right eye (Figure 3A). In his left eye, a large temporal retinoschisis involved the macula (Figure 3B). The parents (II-6, II-7) of the proband did not show any noticeable ocular abnormalities.

By examining all other family members at their local residence, five additional males in the family were diagnosed as having the disease (Table 2). Fundus photographs, however, were not available for these patients. A 35-year-old affected male (II-17) showed foveal stellate cystic change with no peripheral retinoschisis in both eyes. Four affected males (II-10, III-20, III-22, and III-23) showed large peripheral retinoschisis in both eyes. Among these four patients, peripheral retinoschisis involved the macula in five eyes and the other three eyes showed foveal stellate cystic change together with peripheral retinoschisis (Table 2). All other family members showed normal fundus at the time of examination.

### Surgical intervention

A standard three-port pars plana vitrectomy was performed on the left eye of the proband (III-14) under general anesthesia. After careful removal of the vitreous hemorrhage, schitic inner retina, and scar tissues in the vitreous, the macula was found to be schitic with cystic configuration, and only the superonasal retina was relatively normal. Three to four rows of endolaser photocoagulation were applied along the edge of the relatively normal retina, and no intraocular tamponade was implanted. Six months after surgery, the best corrected visual acuity in the left eye increased from hand motion pre-operatively to 0.1, and the retina remained attached (Figure 2E). Best corrected visual acuity in the right eye remained 0.05, and intraocular pressure was normal in both eyes.

### Mutation screening

**Figure 4:**
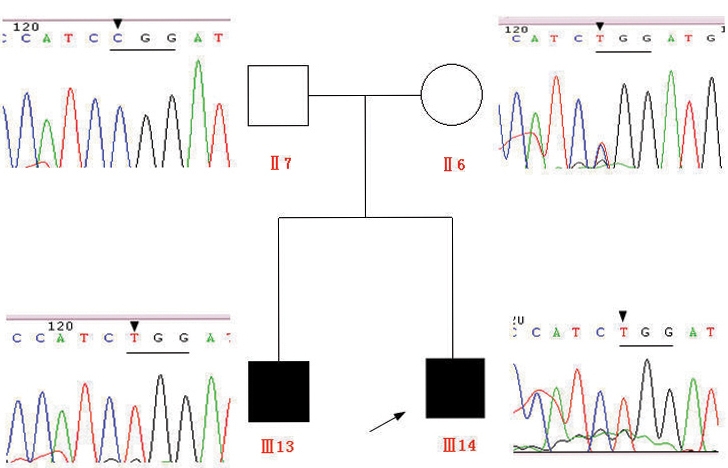
Mutation screening of the retinoschisis 1 gene for the proband, his elder brother, and their parents in the Chinese family with X-linked juvenile retinoschisis. A missense mutation of c.637C>T (R213W) in exon 6 of the retinoschisis 1 (*RS1*) gene was identified for the proband (III-14) and his brother (III-13). The mother (II-6) was heterozygous for the mutation, and the father (II-7) was normal.

All exons of the *RS1* gene were screened for mutation detection in the family. A missense mutation of C to T at position 637 of exon 6 (c.637C>T) was identified by direct sequencing of the PCR products in the proband (III-14) and his elder brother (III-13), which predicted an arginine to tryptophan amino acid substitution at codon 213 (R213W; Figure 4). All other exons were found to be normal. The proband’s mother (II-6), who was clinically normal, showed a heterozygous R213W mutation, and the father (II-7) showed a normal sequence (Figure 4).

**Figure 5:**
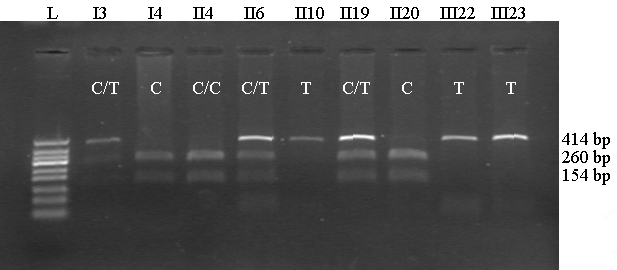
Representative 2% agarose gel of the restriction enzyme digestion analysis using MspI. The enzyme cut the PCR products into two fragments in unaffected males (I-4, II-20) and the normal female (II-4) but could not cut the products in affected males (II-10, III-22, III-23). Female carriers (I-3, II-6, II-19) exhibit a heterozygous status.

All the other 24 family members with genomic DNA available were then analyzed for the R213W missense mutation by restriction enzyme digestion using MspI. The PCR product was digested into two smaller fragments (260 bp and 154 bp) in unaffected individuals and remained undigested (414 bp) in affected individuals (Figure 5). The digestion pattern in female carriers, however, showed three bands, including two digested bands (260 bp and 154 bp) and one undigested band (414 bp), after electrophoresis on the agarose gel (Figure 5). The results from restriction enzyme digestion were further confirmed by direct sequencing of the PCR products in ten key members of the family, including five affected individuals (II-10, II-17,III-20, III-22, and III-23), three female carriers (I-3, I-5, and II-19), and two unaffected individuals (I-4, II-21). The data from the sequencing method were consistent with data from the restriction enzyme digestion method used in all study subjects. Mutation screening results for all 28 family members are given in Table 3.

**Table 3:** Screening of R213W mutation in family members with or without X-linked juvenile retinoschisis

**ID**	**Sex**	**Age**	**Phenotype**	**c.637C>T**
I-3	F	70	Carrier	C/T
I-4	M	73	Unaffected	C
I-5	F	64	Carrier	C/T
II-2	F	46	Unaffected	C/C
II-4	F	42	Unaffected	C/C
II-5	M	45	Unaffected	C
II-6	F	40	Carrier	C/T
II-7	M	42	Unaffected	C
II-10	M	36	Affected	T
II-11	F	34	Unaffected	C/C
II-12	M	31	Unaffected	C
II-13	F	28	Unaffected	C/C
II-17	M	35	Affected	T
II-19	F	37	Carrier	C/T
II-20	M	40	Unaffected	C
II-21	F	35	Unaffected	C/C
III-1	M	31	Unaffected	C
III-4	F	26	Unaffected	C/C
III-6	F	22	Unaffected	C/C
III-10	M	23	Unaffected	C
III-11	F	21	Unaffected	C/C
III-12	F	15	Unaffected	C/C
III-13	M	7	Affected	T
III-14	M	5	Affected	T
III-20	M	18	Affected	T
III-22	M	16	Affected	T
III-23	M	11	Affected	T
IV-1	M	4	Unaffected	C

This table summarizes the screening results of c.637C>T (R213W) mutation in the 28 family members with or without X-linked juvenile retinoschisis. Abbreviations: M represents male; F represents female.

## Discussion

To date, numerous disease-causing mutations in *RS1* have been recognized as genetic entities for XLRS patients in different ethnicities. In this study, we reported a large Chinese family with XLRS, where mutation analysis of *RS1* revealed a missense R213W mutation in all seven affected males and a heterozygous R213W mutation in all four female carriers. The R213W mutation was not identified in other members of the family. While this is the first report of the R213W mutation in Chinese patients, this missense mutation has previously been identified in a Mexican–Spanish family and two Indian patients with juvenile XLRS [15,18]. The R213W mutation is within the phylogenetically conserved discoidin motif that is considered to be critical for RS1 function and predicted to encompass a highly folded globular conformation in the protein retinoschisin [7,19]. It is hypothesized that the missense R213W mutation may interfere with protein folding, leading to an abnormal conformation and intracellular retention of the mutant protein, which causes affected males to have little or no detectable retinoschisin within retina and results in disease phenotypes [2].

Although the same R213W mutation was identified in all seven affected male patients in the reported Chinese family, clinical findings of patients within the family exhibited remarkable phenotypic variability, ranging from foveal stellate cystic change to severe bilateral peripheral retinoschisis and highly elevated bullous retinoschisis with vitreous hemorrhage. All patients reported a reduced visual acuity since early childhood, and we found no obvious correlation between the age of onset and the severity of the disease. In contrast to our patients, affected individuals in the Mexican–Spanish family with the same R213W mutation showed only subtle intraretinal foveal cysts in a spoke-wheel distribution and early macular atrophy of the retinal pigment epithelium, but none of the affected individuals in the Mexican-Spanish family had peripheral retinoschisis [15]. Two Indian patients with the R213W mutation of *RS1* were reported to have foveal and peripheral retinoschisis in both eyes [18]. Comparison of our findings with these previously reported patients of other ethnicities, together with the clinical findings within the reported Chinese family in the current study, suggest that there is no simple phenotype–genotype correlation and that disease severity is not mutation dependent in XLRS. It is therefore apparent that other genetic (polymorphisms) and/or environmental factors are likely to act as significant phenotypic modifiers [13,20,21]. According to Wang et al. [2], the phenotype of XLRS may depend on the secretory capacity of retinoschisin-secreting cells and the degree to which they may induce the unfolded protein response.

The proband of the reported Chinese family was a 5-year-old boy with bullous retinoschisis and vitreous hemorrhage. As vitreous opacities obscured the fundus from detailed examination, diagnosis of retinoschisis for the proband was not confirmed until surgery was done. In clinically suspected cases, full-field electroretinogram (ERG) can be applied, and a negative ERG with a reduced b/a wave ratio (< 1.0) is often considered a key diagnostic feature. However, more than 40% of patients with *RS1* mutations did not have a negative ERG [22]. ERG examination also requires a cooperative patient, a circumstance that cannot always be achieved in young children. Fundus autofluorescence (FAF) and optical coherence tomography (OCT) are additional diagnostic tools for suspected cases and can provide helpful information regarding macular morphology [22,23] but cannot be applied to a patient with severe vitreous opacities. In cases such as the proband of this reported family, molecular genetic analysis of *RS1* will undoubtedly provide an alternative and powerful tool to verify the diagnostic findings for the clinical practice. Identification of the disease-causing mutation in patients with XLRS will also help the family in terms of genetic counseling regarding the prognosis of the disease.

In summary, we have reported a large Chinese family of XLRS with the R213W mutation of *RS1*. Clinical findings of the affected male patients within the family showed varying severity of disease, providing evidence of phenotypic variability in XLRS. Further studies to investigate the genetic and/or environmental factors influencing the phenotypic variability for a defined *RS1* mutation will help to better understand the pathogenesis of the disease and may have important therapeutic implications.
